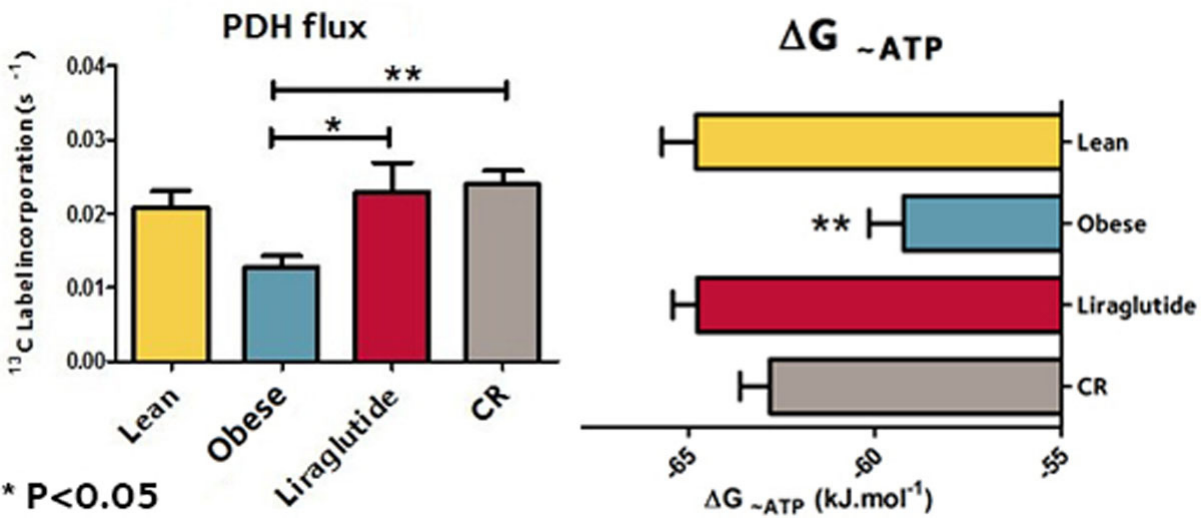# Hyperpolarized ^13^C and ^31^P magnetic resonance spectroscopy identify pyruvate dehydrogenase as a therapeutic target in obesity cardiomyopathy

**DOI:** 10.1186/1532-429X-17-S1-O16

**Published:** 2015-02-03

**Authors:** Andrew J Lewis, Michael S Dodd, Joevin Sourdon, Kieran Clarke, Stefan Neubauer, Damian J Tyler, Oliver Rider

**Affiliations:** 1 Department of Physiology, Anatomy and Genetics, University of Oxford, Oxford, UK; 2 University of Oxford Centre for Clinical Magnetic Resonance Research, Radcliffe Department of Medicine, University of Oxford, Oxford, UK

## Background

Although the link between altered myocardial substrate selection and impaired function in obesity cardiomyopathy is unclear, it is likely to involve altered activity of pyruvate dehydrogenase (PDH), a key regulator of carbohydrate metabolism. We hypothesised that; 1) obesity would impair myocardial carbohydrate oxidation, in turn reducing energetic reserve and predisposing to functional impairment and 2) normalisation of carbohydrate metabolism either pharmacologically or with calorie restriction would reverse these changes. Unlike traditional methods, hyperpolarized [^13^C]pyruvate MR spectroscopy (MRS) enables repeated *in vivo* measurement of both cardiac PDH flux and the first span of the tricarboxylic acid cycle and is therefore an ideal tool to investigate this question.

## Methods

Long Evans rats with high fat diet induced obesity (n=36) and normal weight controls (n=12) were diet standardised. We used *in vivo* hyperpolarized [1-^13^C] and [2-^13^C]pyruvate MRS (prototype hyperpolarizer, 7T MR system) to assess carbohydrate metabolism, echocardiography to assess diastolic function and perfused heart ^31^P-MRS (11.7T) to assess myocardial energetics. Two groups of obese rats were subsequently treated with either calorie restriction (70% usual intake, 28 days, n=12) or a GLP-1 analogue (Liraglutide 0.5mg/kg twice daily, 7 days, n=12) to increase carbohydrate metabolism and restudied.

## Results

Obese animals demonstrated left ventricular hypertrophy and diastolic, but not systolic, dysfunction (E/E' 26±1.5 vs 14±0.86, P<0.05). Hyperpolarized ^13^C-MRS demonstrated that obesity not only dramatically reduced myocardial PDH flux (by 40%, P<0.01) but also increased the normalised rate of ^13^C label incorporation into citrate (by 118%, P<0.05) and glutamate (by 54%, P<0.01) without change in ^13^C label incorporation into lactate or acetylcarnitine (P=ns). Obesity impaired the myocardial energetic state: both the free energy change of ATP hydrolysis and PCr/ATP ratio were significantly impaired (by 5% and 13%, respectively, P<0.05 for both). Obesity significantly increased tissue PDK4 levels (P<0.05), the major regulator of cardiac PDH. Liraglutide treatment (weight neutral, P=ns versus the same group pre-treatment) and calorie restriction (leading to 15% weight loss versus age matched controls, P<0.001) normalised PDH flux, TCA cycle metabolism, myocardial energetics and function (all P<0.05).

## Conclusions

In a rodent model of obesity, hyperpolarized ^13^C-MRS identified abnormal cardiac metabolic substrate selection which was associated with abnormal TCA cycle metabolism, impaired high energy phosphorous metabolism and functional deficit. Normalisation of PDH flux with either Liraglutide or calorie restriction reversed this energetic deficit and normalized function, suggesting PDH may be a novel therapeutic target.

## Funding

The Academy of Medical Sciences, British Heart Foundation.

**Figure 1:**